# HSIL-Based Synthesis of Ultracrystalline K,Na-JBW, a Zeolite Exhibiting Exceptional Framework Ordering and Flexibility

**DOI:** 10.1021/acs.chemmater.2c01059

**Published:** 2022-06-16

**Authors:** Karel Asselman, Sambhu Radhakrishnan, Nick Pellens, C. Vinod Chandran, Maarten Houlleberghs, Yijue Xu, Johan A. Martens, Sreeprasanth Pulinthanathu Sree, Christine E.A. Kirschhock, Eric Breynaert

**Affiliations:** † Center for Surface Chemistry and Catalysis − Characterisation and Application Team (COK-KAT), KU Leuven, Celestijnenlaan 200F, 3000 Leuven, Belgium; ‡ NMR/X-ray Platform for Convergence Research (NMRCoRe), KU Leuven, Celestijnenlaan 200F, 3000 Leuven, Belgium; § National High Magnetic Field Laboratory, 1860 East Paul Dirac Drive, Tallahassee, Florida 32310, United States

## Abstract

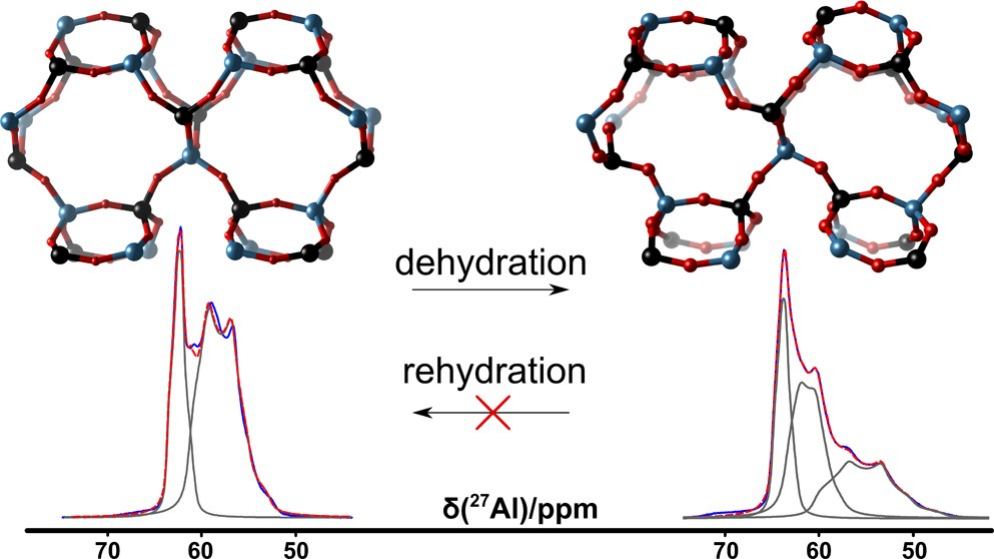

A reproducible synthesis strategy for ultracrystalline K,Na-aluminosilicate JBW zeolite is reported. The synthesis uses a Na-based hydrated silicate ionic liquid (HSIL) as a silicon source and gibbsite as the aluminum source. ^27^Al and ^23^Na NMR spectra exhibit crystalline second-order quadrupole patterns in the hydrated as well as dehydrated states and distinct resonances for different T-sites demonstrating an exceptional degree of order of the elements of the JBW framework, observed for the first time in a zeolite. Detailed structural analysis via NMR crystallography, combining powder X-ray diffraction and solid-state NMR of all elements (^27^Al, ^29^Si, ^23^Na, ^39^K, and ^1^H), reveals remarkable de- and rehydration behavior of the JBW framework, transforming from its as-made hydrated structure via a modified anhydrous state into a different rehydrated symmetry while showing astonishing flexibility for a semicondensed aluminosilicate. Its crystallinity, exceptional degree of ordering of the T atoms and sodium cations, and the fully documented structure qualify this defect-free K,Na-aluminosilicate JBW zeolite as a suitable model system for developing NMR modeling methods.

## 1 Introduction

**Figure 1:**
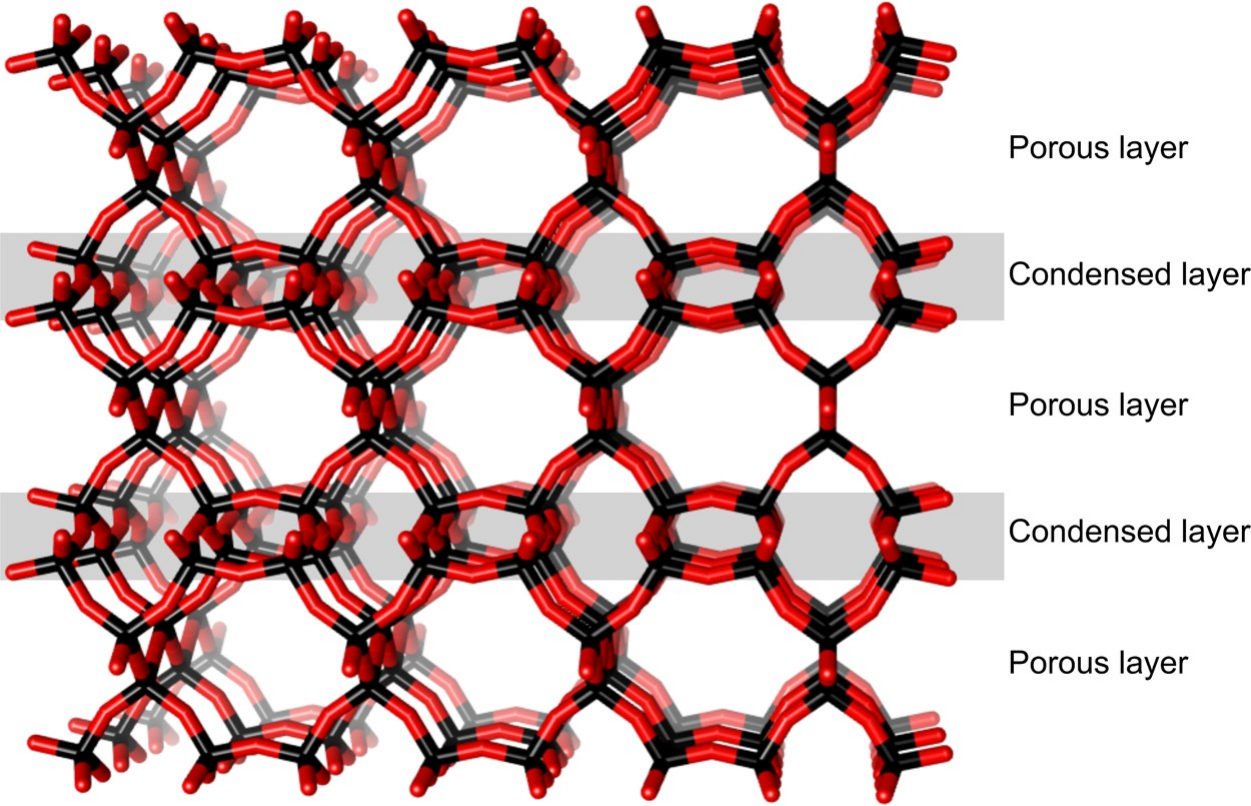
Schematic view of JBW framework, showing the single and double zigzag chains, one-dimensional 8R channels separated by anhydrous, dense layers of collapsed channels of interconnected 6R.

JBW zeolite was named after the Linde type J material and the initials of Barrer and White, who first reported it, referring to it as nepheline hydrate.^1^ Its structure was solved only 30 years later by Hansen and Fälth^2^ in 1982. The aluminosilicate framework in JBW consists of alternating double and single zigzag chains along the *c* direction (Figure 1). They are sideways connected, forming one-dimensional asymmetric 8R channels with dimensions 4.8 × 3.8 Å, separated by layers of collapsed 6R pores, containing solely anhydrous sodium cations. Due of this feature, JBW is counted among the semidense zeolites, on the borderline between porous zeolites and dense oxides. The anhydrous 6R layers stabilize the high alumina framework by strong interactions between the framework and the cations.^3^ Early syntheses of Na-JBW frequently suffered from impurities, analcime and cancrinite being reported as common side phases.^1,2,4^ Synthesis of phase-pure JBW was first reported by Healey et al., who also reported a detailed structural analysis of this phase by powder neutron diffraction.^5^ It was proposed that the presence of potassium next to sodium cations is a prerequisite for the formation of phase-pure JBW. This assumption is based on an interesting cation distribution, where the cation types occupy distinct sites in the zeolite framework. Anhydrous sodium cations are located in the semicondensed layer, while hydrated potassium resides in the 8R pores.^3,5^ The reported synthesis required the transformation of meta-kaolin at high temperatures (225 °C), with moderate yield.

*In silico* experiments have suggested that the JBW topology in its pure siliceous form is suitable for CO_2_ capture. It exhibited the highest isosteric heat of CO_2_ adsorption among 37 studied topologies, with the highest selectivity by far for CO_2_ in CO_2_/N_2_ gas mixtures.^6,7^ Simulated ad- and desorption isotherms showed for JBW the lowest parasitic energies for CO_2_ of all investigated zeolite topologies.^8^ However, owing to the unavailability of JBW in high-silica form, this could not be confirmed experimentally. Furthermore, computational adsorption studies, for simplicity, typically treat the zeolite framework as rigid scaffolds, to be emptied and redecorated at will with guest molecules to examine ad- and desorption. In reality, porous materials like zeolites crystallize with inorganic or organic cations and solvent molecules occupying the voids. Removal or replacement of either of the pore-filling species impacts the framework stability and often also its geometry. As a consequence, important properties like diffusivity, selectivity, or adsorption energy need to account for the exact state of the zeolite during the process.^9^

This work reports a straightforward, reproducible method to quantitatively convert generic silicate sources into high quality aluminosilicate JBW zeolite at moderate temperatures (150 °C). We revisit the crystal structure of JBW and describe unusual structural changes of this material upon drying and rehydration via a complete structural characterization through NMR crystallography, combining solid-state NMR spectroscopy of all elements (^27^Al, ^29^Si, ^23^Na, ^39^K, and ^1^H) and X-ray diffraction, using Rietveld refinement.

## 2 Experimental Section

The here reported synthesis of JBW zeolite uses a modified hydrated silicate ionic liquid protocol.^10−12^ A sodium-based hydrated silicate ionic liquid (HSIL) is prepared by mixing NaOH (Fischer Scientific, 98+%), H_2_O, and TEOS (Acros Organics, 98%) in 1.5:1.0:25 molar ratios. Complete hydrolysis of TEOS leads to spontaneous phase separation, yielding a biphasic system with the Na-HSIL (1 SiO_2_/1.5 NaOH/4.3 H_2_O) as the dense bottom phase. The Na-HSIL was mixed with KOH pellets (Fischer Scientific, 85%), aluminum hydroxide (VWR, technical grade Gibbsite), and water to obtain a final synthesis mixture with molar composition 1 SiO_2_/0.5 Al_2_O_3_/1.5 NaOH/0.7 KOH/27 H_2_O. After vigorous stirring for 1 h, the mixture appears as a homogeneous suspension of partially undissolved Al(OH)_3_ particles. The sample was incubated in a Teflon-lined stainless-steel autoclave in a rotating oven at 150 °C for 7 days. Afterward, crystallites were recovered by centrifugation and repeatedly rinsed with distilled H_2_O until the supernatant was pH-neutral and subsequently dried at 60 °C. High-resolution SEM images were recorded on a Nova NanoSEM450 (FEI, Hillsboro, OR). To study the effects of dehydration, samples were subjected to high vacuum conditions (1 mbar) for 16 h at 200 °C and sealed afterward to prevent rehydration by ambient moisture. Laboratory high-resolution PXRD patterns (Cu Kα_1_ radiation) were recorded at room temperature on a STOE STADI MP diffractometer with a focusing Ge(111) monochromator in Debye–Scherrer geometry, with a linear position sensitive detector (internal resolution 0.01°). Absorption corrections for Debye–Scherrer geometry were applied, and profile fitting and Rietveld refinement were performed in the GSAS package.^13^ The background was fitted using a shifted Chebyshev polynomial; peak profiles were described with a pseudo-Voight type function (GSAS profile function type 4). Scattering factors of the neutral elements were used for framework atoms and cations, and the scattering factor of O^2–^ was used for water. Si and Al contents were determined from dissolved samples on an axial simultaneous ICP-OES instrument (Varian 720-ES) with a cooled cone interface and oxygen-free optics. Na and K contents were determined via atomic absorption spectroscopy (Varian SpectrAA 20 Plus). Thermogravimetric analysis was performed on a TGA Q500 (TA Instruments) under a N_2_ flow (10 mL/min) with a heating rate of 2 °C/min between 25 and 850 °C. ^27^Al and ^1^H solid-state NMR experiments were performed on a Bruker Avance III 500 MHz NMR spectrometer (9.4 T) equipped with a 4 mm H/X/Y solid state MAS probe. Larmor frequencies were 130.52 and 500.87 MHz for ^27^Al and ^1^H, respectively. The samples were filled in a 4 mm ZrO_2_ rotor and spun at 15 kHz MAS frequency. ^1^H NMR spectra were acquired with an 83 kHz RF pulse, recycle delay of 5 s, and eight transients. A total of 1024 scans (recycle delay of 2 s) were recorded for ^27^Al MAS NMR with ^1^H decoupling using the SW_f_-SPINAL method.^15^ The RF strengths used for ^27^Al and ^1^H for decoupling are 150 kHz and 55 kHz, respectively. ^27^Al spectra were recorded using a 15° flip angle. For Z-filtered ^27^Al MQMAS measurements, 600 slices in the indirect dimension were acquired with a t1 increment of 33.33 μs, a relaxation delay of 2 s, and 60 transients in the direct dimension. ^29^Si MAS NMR experiments were carried out on a Bruker Avance III 300 MHz NMR spectrometer (7.1 T) equipped with a 4 mm H/X solid-state MAS probe. The ^29^Si Larmor frequency was 59.62 MHz. The MAS frequency used was at 10 kHz. The RF strengths used for ^29^Si excitation and ^1^H decoupling were 66 and 40 kHz, respectively. A total of 320 transients were collected with 600 s of recycle delay. ^23^Na measurements were performed on a Bruker Ascend 800 MHz (18.8 T) equipped with a 1.9 mm H/X/Y probe. The sample was filled in a 1.9 mm ZrO_2_ rotor and spun at 30 kHz. The RF strengths used were 110 kHz on ^23^Na and 19 kHz on ^1^H for decoupling. ^29^Si chemical shifts were referenced to a secondary reference, Q_8_M_8_, which was further referenced against a primary reference, tetramethylsilane (TMS). ^27^Al chemical shifts were referenced against 0.1 M solutions of Al(NO_3_)_3_. ^23^Na spectra were referenced to 0.1 M NaCl solution in D_2_O. All of the ^39^K MAS NMR experiments were carried out on a Bruker 830 MHz NMR spectrometer at the National High Magnetic Field Laboratory (Tallahassee, Florida) operating at a 19.6 T magnetic field and with a ^39^K Larmor frequency of 38.80 MHz. The samples were packed in 3.2 mm zirconia rotors and spun up to 20 kHz. QCPMG (Quadrupolar Carr–Purcell–Meiboom–Gill) signals with 9 to 12 echoes for the three samples were acquired, yielding the spikelet pattern after Fourier transformation. The excitation and refocusing pulses for the QCPMG sequence are 4 and 8 μs, respectively. A range of 247 000 to 1 835 000 transients were recorded for all experiments. A WURST (Wideband-Uniform Rate-Smooth Truncation) pulse lasting 32 rotor periods (1.6 ms) with a 200 kHz offset and a nutation frequency of 28 kHz was applied to the satellite transitions to enhance the central transition polarization prior to the QCPMG. The ^39^K experiments used the ^17^O NMR resonance of aqueous D_2_O as a secondary reference using the ^39^K/^17^O spectrometer frequency (SF) interconversion: SF(^39^K) = SF(^17^O) × 4.666373/13.556457 (frequency ratios calculated from the magnetogyric ratios^14^).

## 3 Results

### 3.1 As-Made, Hydrated JBW

**Figure 2:**
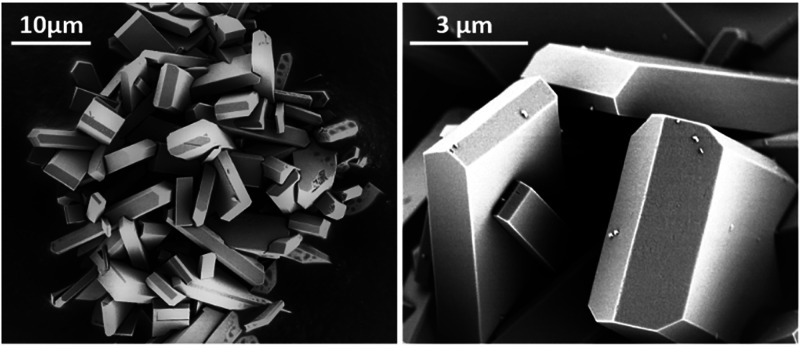
Scanning electron microscopy images of as-synthesized JBW crystals.

SEM imaging (Figure 2) reveals polydisperse, elongated, flattened prismatic crystals with a maximum length up to about 20 μm. This crystal habit is typical for previously reported JBW zeotypes.^2,4,15^ No side phases were detected. Both ICP analysis and ^29^Si NMR indicated a Si/Al ratio of unity, implying a perfect framework Si–Al alteration as imposed by Löwenstein’s rule. Quantitative ^1^H NMR analysis^16^ yielded a value for the water content of 4.04 wt %, consistent with thermogravimetric analysis, which shows a 4.36 wt % water loss between RT and 800 °C (Figure S1). The Na/K ratio, as determined by AAS, was 2.31, approximating to an ideal chemical composition of K_1_Na_2_[SiAlO_4_]_3_·H_2_O (Table S1).

**Figure 3:**
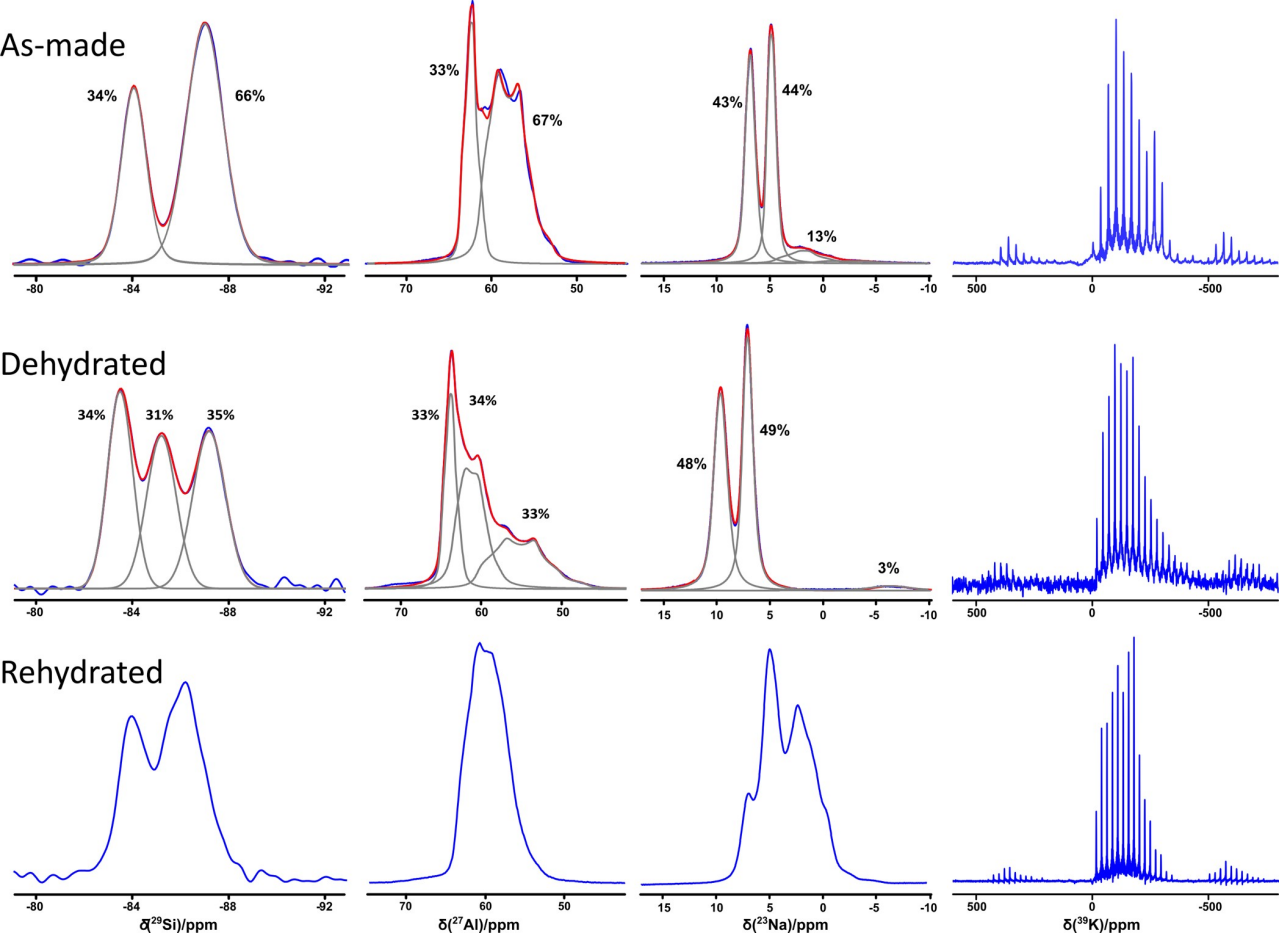
^29^Si, ^27^Al, ^23^Na, and ^39^K NMR spectra of as-made, dehydrated, and rehydrated JBW.

**Figure 4:**
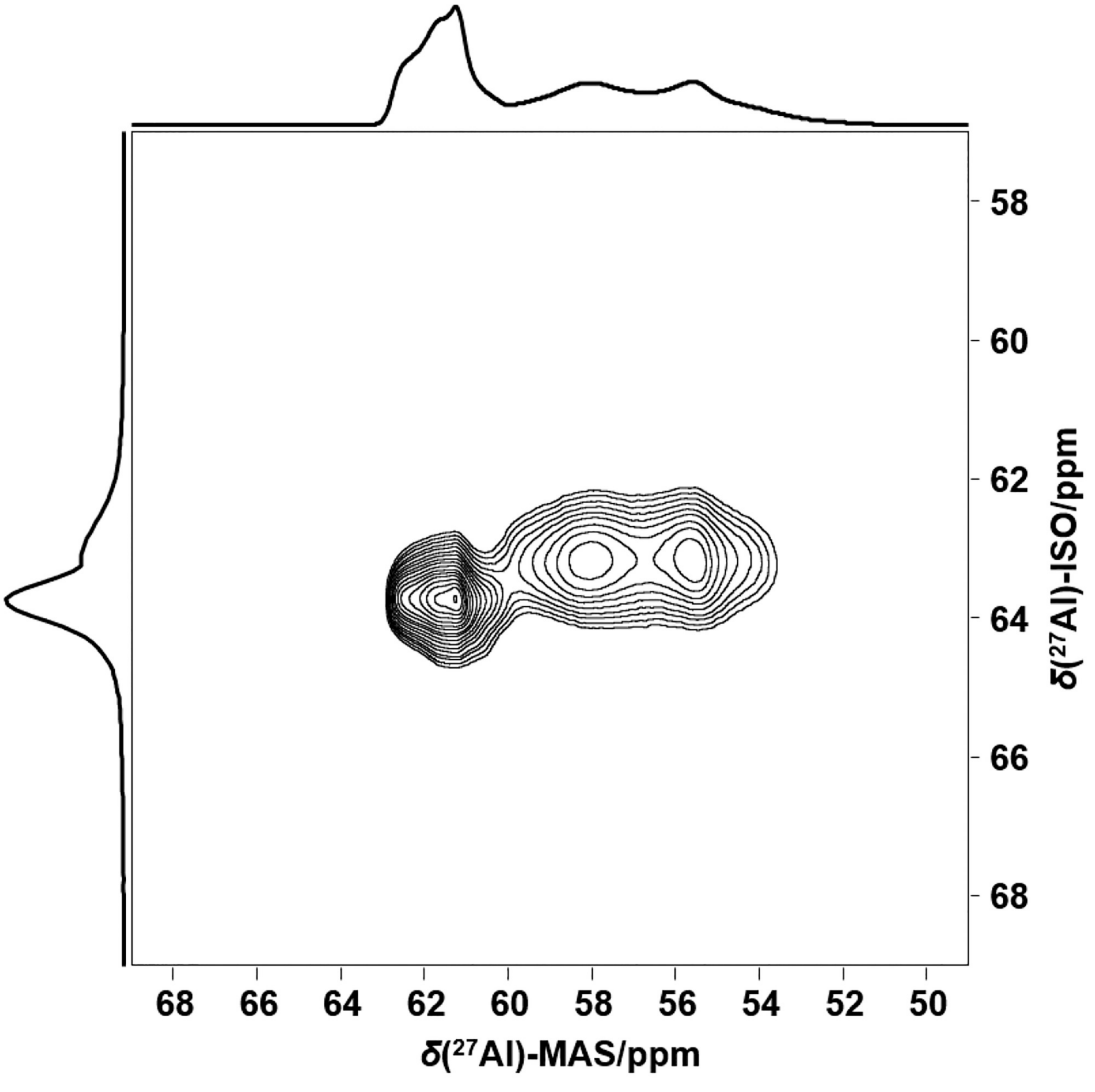
^27^Al 3Q-MAS NMR spectrum of as-made K,Na-JBW.

The direct excitation ^29^Si MAS NMR spectrum of JBW exhibits two symmetrical resonances centered at −84 ppm and −87 ppm corresponding to two crystallographically unique Si sites (Figure 3). Spectral decomposition revealed a relative site population of 2:1. Also, in the 1D ^27^Al MAS spectrum, and confirmed by 2D ^27^Al 3QMAS NMR, two distinct Al signals corresponding to tetrahedral Al can be discerned (Figures 3 and 4). Spectral decomposition confirms the same relative occupancy (2:1) as Si, with isotropic chemical shifts of δ_iso_ = 63.7 and 61.7 ppm, respectively (Figures 3 and 4). Uniquely, the two ^27^Al resonances exhibit crystalline second-order quadrupolar patterns with quadrupolar parameters *C*_Q_ = 1.8 and 3.2 MHz and η_Q_ = 0.69 and 0.39, respectively (Table S8). This is a remarkable observation, as zeolites typically exhibit unresolved Al signals resulting from a distribution of the electric-field gradients around ^27^Al as described by the Czjzek model.^17,18^ The occurrence of crystalline second-order quadrupole patterns for ^27^Al demonstrates that an exceptional degree of ordering of the local symmetry is present in JBW zeolite, unlike what is typically observed in other zeolites. ^23^Na NMR also revealed the presence of two major resonances in a 1:1 ratio. Also here, second order quadrupolar line shapes with *C*_Q_’s of 1.1 and 1 MHz and η_Q_’s of 0.62 and 0.47 were observed. A broadened shoulder in the spectrum, integrating to approximately 13% of the measured intensity, was identified to belonging to a dehydrated and subsequent rehydrated fraction of the sample (see section 3.3), either during the 60 °C drying procedure after washing or during the hydrothermal synthesis itself. Presumably, these signals originate from the outermost crystal layers that are dehydrated first, explaining their absence in the diffraction pattern. Contrary to the ^29^Si, ^27^Al, and ^23^Na NMR spectra, for which the individual site contributions are readily quantified, the ^39^K spectrum shows more complexity (Figure 3). The spectral envelope could not be reliably reconstructed from the spikelet patterns (Figure S7), and the spectra reveal no clearly defined contributions from individual potassium sites, indicating that the environment for potassium in JBW is more distributed than for the other elements.

Indexing of the diffraction pattern revealed an orthorhombic unit cell with dimensions *a* = 15.142 Å, *b* = 8.126 Å, and *c* = 5.176 Å. Systematic absences indicated space group *Pmn*2_1_. Several structural studies on JBW have reported a possible doubling of the lattice in the *b* direction. Following an appropriate unit cell transformation, this lowers the symmetry to *Pna*2_1_.^2,5^ In the present sample, however, there is no evidence for doubling of the lattice, and intensity extraction in the lower symmetry *Pna*2_1_ cell did not result in improved profile fits. In *Pmn*2_1_, JBW has two crystallographic sites with a relative multiplicity of 2:1 for Si and Al, in full agreement with the NMR analysis. Refinement, therefore, proceeded in this space group. The structure reported by Hansen and Fälth^2^ was used as a starting model for the framework atoms, after conversion of the coordinates (reported in the *Pna*2_1_ structure) to the smaller unit cell with higher symmetry *Pmn*2_1_. Difference Fourier analysis revealed the presence of two sodium atoms in the anhydrous layer, on their expected positions. Coordinates and occupancies were refined, converging to values of 0.992 and 1.014, respectively. When these values were correlated with the signal intensities and perfect local symmetry derived from ^23^Na NMR, they were consequently fixed at 1. Healey et al.^5^ described chains of alternating potassium and water in space group *Pna*2_1_, where only 50% of the water sites were occupied (H_2_O/K = 0.5). In a comparison to our own quantification from TGA and quantitative ^1^H NMR, we conclude that the water content is twice as high, with a 1-to-1 ratio of potassium and water. In *Pmn*2_1_ symmetry, each 8R channel is symmetrically equivalent, with a short *c*-axis leading to an overlap of water and potassium sites. Both species were added to the structure model at regions with high electron density of the difference Fourier map, and subsequently their positions were refined. The occupancy factor of potassium was fixed at 0.5, complying with the sample stoichiometry. In the final cycles, positional and temperature factors of all atoms were refined simultaneously, leading to convergence with reliability factors of *R*_wp_ = 5.95% and *R*_F2_ = 4.54%. Final structure coordinates, refinement parameters, and bond lengths and angles are listed in Tables S2 and S6. The final structure closely resembles that of the reported Na_2_Rb[AlGeO_4_]_3_·H_2_O aluminogermanate JBW structure.^19^ As potassium and water are crystallographically located at virtually the same position in the pores, the 8R channels in this structure necessarily contain chains of alternating potassium and water, as two adjacent sites are too closely spaced to both be occupied by K^+^. Between neighboring 8R channels, however, no correlation between respective potassium and water positions is detected, resulting in the here-described distribution.

Considering the perfect ordering of framework and sodium atoms, it was tested whether an ordered potassium distribution could describe the structure with higher accuracy. Ordered distributions for potassium (unique crystallographic sites with full occupancy) require the structure to be described in a different space group. The symmetries that give such an ordered K distribution and still comply with the observed number of crystallographic sites and multiplicities of Si, Al, and Na determined via NMR are *P*2/*n*, *P*2_1_/*m* in a monoclinic setting, or *Pccn* in an orthorhombic cell, with a doubled *a* lattice. Refinement in either of these settings did not converge. Therefore, we conclude that the original refinement in *Pmn*2_1_ is the best setting that adequately describes the measured data. This suggests that K and H_2_O are truly distributed over 2 equiv sites. Their high thermal displacement factor relative to sodium and framework atoms furthermore indicates water and potassium either have static or dynamic disorder around these sites. (Table S2). This is consistent with ^39^K NMR, which is the only spectrum that cannot be reliably decomposed in its individual contributions.

### 3.2 Structure Changes upon Dehydration

**Figure 5:**
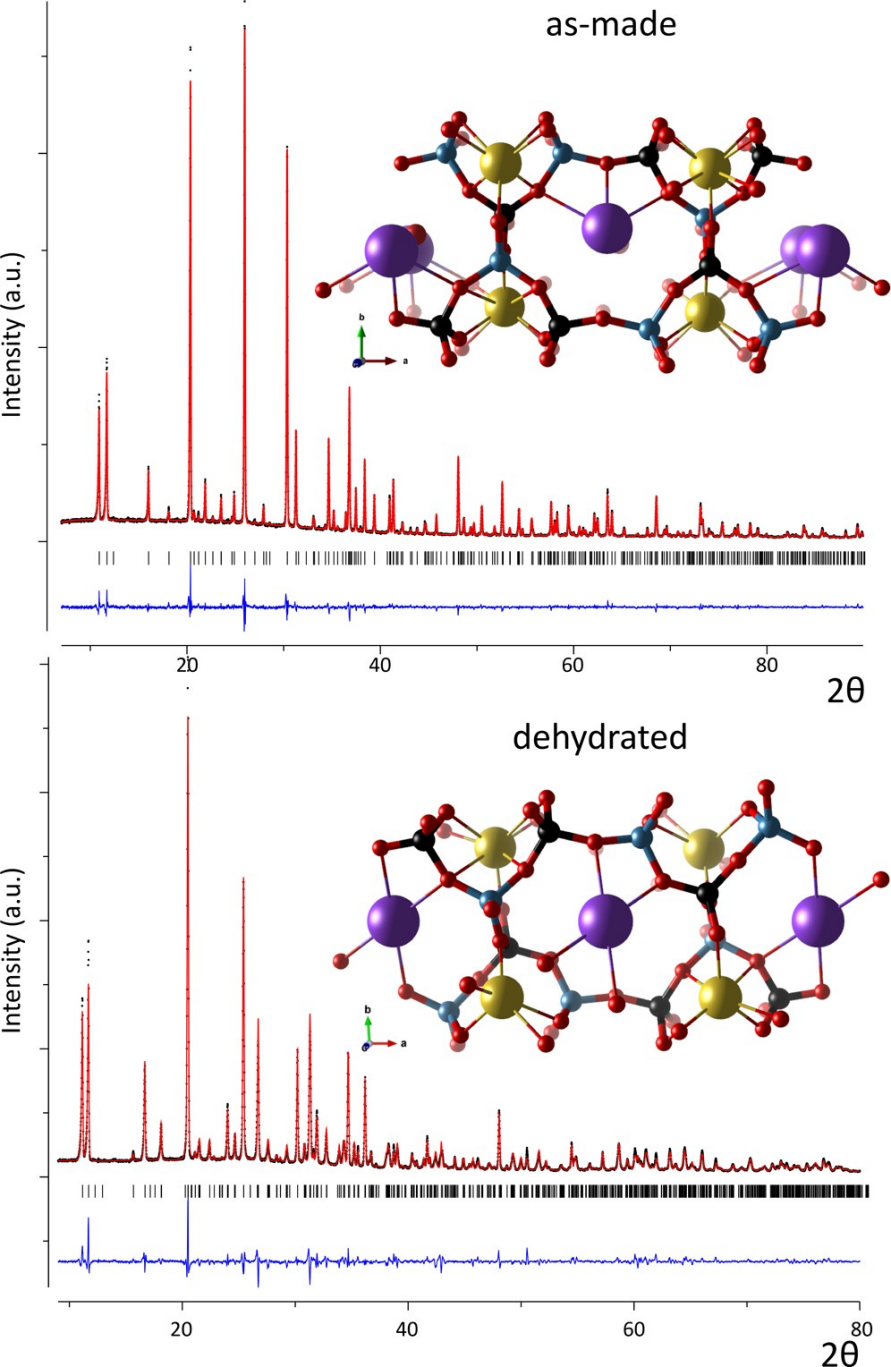
Rietveld refinement plot and visualization of as-made (top) and dehydrated (bottom) JBW. The blue line is the difference between observed (black) and calculated (red) intensities. The inserts visualize the refined configurations of the framework and Na (yellow), K (violet), and H_2_O (red) atom positions.

**Figure 6:**
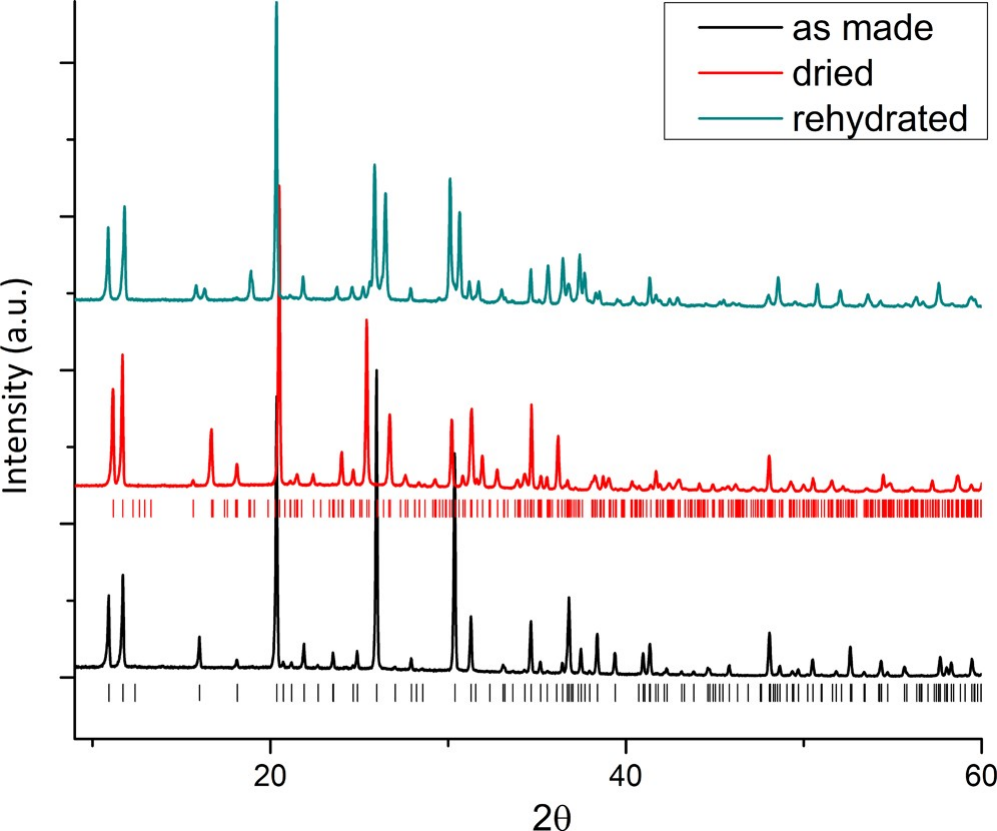
Diffraction patterns of JBW samples in the native state and after de- and rehydration.

The structural importance of water in the JBW framework was evaluated by dehydrating the crystals under high vacuum conditions (1 mbar) at 200 °C for 24 h. ^1^H NMR showed that this treatment fully dehydrates the sample without introduction of defects in the crystals, as resonances for Si–OH or Al–OH were not detected (Figure S5). ^29^Si and ^27^Al NMR indicate remarkable structural changes, now showing three distinct Si resonances as well as three distinct crystalline ^27^Al second-order quadrupole patterns, occurring in a virtually 1:1:1 ratio. The ^23^Na NMR spectrum still shows two contributions in a 1:1 ratio strongly shifted downfield compared to the hydrated structure (Figure 3). This indicates a reduction in symmetry, where the Si and Al located on general positions in the as-made structure in space group *Pmn*21 split into two crystallographically independent sites. Accordingly, the diffraction pattern is strictly different from the hydrated sample (Figures 5 and 6). The pattern was initially indexed as monoclinic with lattice constants *a* = 7.94 Å, *b* = 5.17 Å, *c* = 15.17 Å, and monoclinic angle β = 93.63°. However, full profile fitting revealed that the cell symmetry is actually triclinic, with refined lattice constants *a* = 15.165 Å, *b* = 7.946 Å, *c* = 5.169 Å, α = 89.948°, β = 89.848°, and γ = 93.613° (unit cell directions chosen to align with crystallographic directions of the as-made sample). In this cell, with space group *P*1̅, there are three Si and Al sites and two Na sites with equal multiplicity, fully consistent with the ^29^Si, ^27^Al, and ^23^Na NMR analysis. Refinement therefore proceeded in this space group. The observed reduction of symmetry upon dehydration renders the structure of the dehydrated JBW complex, with 22 independent scatterers. Considering the good quality of the diffraction data, and the retention of perfect framework ordering of the sample as confirmed by ^29^Si and ^27^Al NMR, a structure refinement of the dehydrated sample was attempted, accounting for *a priori* information from NMR. As a starting model, water was removed from the parent structure, and subsequently the structure was optimized in the new unit cell and space group in GULP^20^ using the Catlow library potentials. Two independent K atoms were placed on the inversion centers in the 8R channel. Soft constraints were imposed on the tetrahedral framework (1.614 ± 0.02 Å and 1.735 ± 0.02 Å for Si–O and Al–O bonds, 2.633 ± 0.1 Å and 2.833 ± 0.1 Å for the O–O distance of SiO_4_ and AlO_4_ tetrahedra, respectively). Inspection of the Fourier map showed that the electron density around K in both 8R channels was more accurately modeled by shifting K slightly away from the inversion center, allowing a split but overlapping site with 50% occupancy. Like in the hydrated structure, potassium shows some positional distribution and dynamics. In the final cycles, all atomic parameters were simultaneously refined resulting in agreement factors *R*_wp_ = 9.06% and *R*_F2_ = 9.99%. The Rietveld plot and crystal structure are visualized in Figure 5. Atomic position parameters are listed in Table S3.

Upon dehydration, the single zigzag chains separating adjacent 8R channels are rotated, resulting in a diagonal contraction of the channel, a shortening along the *b* axis, and a relaxation of the lattice angles (Figure 5, Table 1). The unit cell volume shrinks by 2.40%, paired with a significant distortion of the framework. This is a remarkable change considering the total water content is only 4.04 wt %. The lattice deformation demonstrates that the framework is very flexible, despite being classified as a semicondensed tectosilicate,^3^ confirming earlier theoretical predictions for flexibility of the JBW topology.^21^

**Table 1:** Lattice Parameters and Symmetry of As-Made, Dry, and Rehydrated JBW

	space group	*a* (Å)	*b* (Å)	*c* (Å)	α (deg)	β (deg)	γ (deg)
as-made	*Pmn*2_1_	15.142	8.126	5.176	90	90	90
dehydrated	*P*1	15.165	7.946	5.169	89.948	89.848	93.613
rehydrated	unknown	15.004	16.293	5.176	89.976	89.904	91.741

Potassium loses two coordination partners when water is removed from the channels. By framework contraction, this loss is in part compensated for by a stronger interaction with framework oxygen, resulting in the here-observed deformation (Figures 5 and S6). This effect was demonstrated recently in the extraordinarily flexible GIS framework, which distorts upon dehydration and restores its configuration upon rehydration or CO_2_ adsorption,^9^ as cations relax back to their original, hydrated coordinative state. The degree of deformation was shown to depend on cation type, the effect decreasing for larger cations with lower oxophylicity.

In small pore zeolites, isolated water molecules like in the present structure are well positioned to hydrogen-bond with the zeolite framework, stabilizing the structure.^5,22,23^ The loss of this coordinative stabilization by the water molecules may also contribute to the contraction of the framework. The importance of hydration water on the cation positions and elastic properties of aluminosilicate zeolites has also been demonstrated computionally for a number of topologies and cation types.^24^

Given that, in JBW, sodium ions are fully coordinating to framework oxygen and not to any water in the condensed layer of the parent structure, a significant change in coordination behavior of Na was not expected upon removal of H_2_O. However, in the dehydrated structure, the average Na–O bond length is considerably shortened in the dried JBW compared to the parent structure (Tables S6 and S7, Figure S6), evidenced also by the strong downfield shift of both ^23^Na resonances, indicating a more intimate sodium–framework interaction (Figure 3). This may be caused indirectly by the loss of water molecules originally stabilizing the 8R channels.

### 3.3 Rehydration

**Figure 7:**
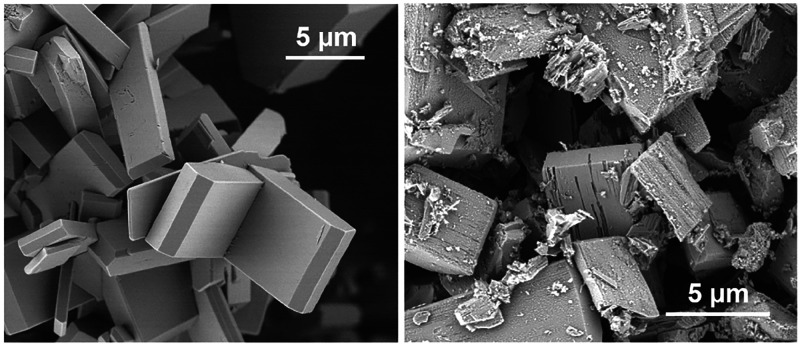
SEM images of JBW after rehydration under mild conditions (left) and after refluxing (right).

Similarly to the reversible de- and rehydration process in GIS,^9^ it was expected that JBW would restore its configuration upon rehydration. The dehydrated JBW sample was rehydrated by the addition of a stoichiometric amount of water in a closed vessel and moderately heated at 60 °C. The sample readily and quantitatively readsorbs its original water content, but the ^1^H NMR spectrum is broader compared to the as-made sample, indicating a broader distribution of water sites (Figure S5). Unexpectedly, the structure does not relax to the as-made form, despite having the same stoichiometry, but is distinct from both as-made and dehydrated structures. Instead, a considerable symmetry reduction is apparent from the ^23^Na, ^29^Si, and ^27^Al NMR spectra, showing complex shapes composed from multiple resonances that could not be properly resolved (Figure 3). Dehydrated JBW, upon readsorbing its original water content, thus incompletely relaxes to its original state. The corresponding diffraction pattern corroborates with the complexity indicated by NMR, showing significant differences in relative peak positions and intensities from both the parent and dehydrated samples, while still being highly crystalline and indicative of a single phase (Figure 6). SEM imaging showed that the crystals were not damaged or destroyed by the applied de- and rehydration cycles (Figure 7). The diffraction pattern was satisfactorily fitted as triclinic with lattice constants *a* = 15.004 Å, *b* = 516.293 Å, *c* = 5.176 Å, α = 89.976°, β = 89.904°, and γ = 91.741°, i.e., an intermediate distortion between the dehydrated and as-made structure (Figure S2). Remarkably, a doubling of the lattice in the *a* direction is necessary to fit the diffraction pattern, as multiple strong peaks remain unindexed in the small cell. We confirmed that full rehydration and partial relaxation upon exposure to humidity happens fast, even when only exposed to ambient room temperature conditions for several hours, but even prolonged exposure to moisture and moderate heat in a closed system does not reinstate the original structure. Due to the apparent complexity of the sample and ambiguous symmetry, refinement of the rehydrated sample remained inconclusive.

Full restoration of the framework was ultimately attained for a fraction of the sample by immersing the dehydrated crystals in excess H_2_O and refluxing at the boiling point for longer than 12 h. Diffraction patterns and NMR spectra, showing the sample to be a mix of the original (as-made) and rehydrated JBW prior to refluxing, are added to the Supporting Information (Figures S3 and S4). However, SEM analysis revealed that the reflux treatment damages and ultimately destroys the crystals (Figure 7). Restoration of the sample to its original state is thus accompanied by at least partial destruction or dissolution of the material.

## 4 Discussion

According to the literature, it is difficult to reproducibly synthesize phase pure JBW.^2,4,5^ The use of HSILs as a Si source next to crystalline Al(OH)_3_ as described in this work, however, yields JBW in high quality and quantity. The procedure is robust and reproducible and appears to be only weakly dependent on synthesis time and temperature. Varying the time and temperature between 4 and 7 days and 140 and 170 °C did not result in significant differences in synthesis outcome. Evaluating the synthesis using alternative silicate sources to HSIL, i.e., waterglass (sodium silicate), colloidal silica solutions (stabilized with sodium), or sodium aluminosilicate, was unsuccessful. Consequently, it seems that the highly ionic HSIL^11,12^ is essential for this synthesis. Up to now, the highly ionic, monophasic HSIL-based syntheses have been demonstrated to crystallize exceptionally ordered zeolites at very low Al supersaturation.^12,25−28^ The current synthesis protocol for JBW however requires that aluminate solubility is greatly exceeded, representing the first HSIL-based synthesis at high Al supersaturation but exhibiting the same advantages as observed at low supersaturation.

In the absence of sintering or the occurrence of other changes to the zeolite framework connectivity during dehydration, zeolites typically exhibit full restoration of their symmetry upon full rehydration of the dehydrated framework. Consequently, also for JBW, full restoration of the as-made symmerty upon rehydration was expected, as K should be able to readily restore its previous coordination environment. Instead, upon rehydration, the framework converts to an alternate local energy minimum preventing reconversion to the as-made structure. We hypothesize sodium to be the culprit, inhibiting framework restoration by its increased interaction with the framework in a dehydrated state. As elaborated in section 3.2, sodium–framework distances are shortened after sample dehydration. This can be visualized by comparison of the partial pair density functions of sodium and framework oxygen, as calculated from the refined crystal structures (Figure S6). The most optimal Na–O bond length in aluminosilicates is approximately 2.3–2.4 Å, and the average Na–O bond length is closer to this value in the dehydrated sample, implying a more favorable coordination environment for sodium. As mentioned previously, a shoulder in the ^23^Na NMR spectrum of the as-made sample (Figure 3) indicates that a fraction of the sodium, approximately 13%, is found in the same state as in the rehydrated sample. It was also verified that the NMR signature of this sodium can be discerned even for a freshly synthesized sample after washing and drying at room temperature in ambient humidity for a week, indicating that a fraction of the sodium in the structure is already in this state during crystallization. Since sodium cations are located in a region of the framework inaccessible to water, rehydration will only affect the hydration state of K. Sodium, being strongly coordinated by the dehydrated framework, would have to increase its energy to restore its original configuration. This implies an energetic barrier, so that relaxation back to the original geometry does not occur under moderate hydrating conditions. Instead, framework restoration to the as-made state requires high temperatures under excess water conditions, where the framework also suffers from dissolution–reprecipitation as a result of increased solubility.

Indexing the powder pattern of the rehydrated sample necessitates at least doubling of the lattice in the *a* direction (Figure S2), the reason being presently unclear. Interestingly, pure Na aluminosilicate JBW has invariably been reported with a doubled cell.^2^ Similarly, K,Na-JBW synthesized at a much higher temperature of 225 °C is reported with a doubled lattice.^5^ That sample was also dried *in vacuo* after synthesis in a desiccator prior to exposure to ambient conditions. Whether this sample underwent dehydration and subsequent rehydration, or if at the high synthesis temperatures the low symmetry state formed directly, remains unclear. In any case, it stands to reason that the resulting structure resembles the “rehydrated” state from our study, rather than the “as-made”, high-symmetry structure. We further propose that the cooperative stabilizing effect of potassium and isolated water molecules in the 8MR channel are required to prevent sodium in the dehydrated layer from distorting the framework and lowering the symmetry during or after crystallization. Potassium has a higher affinity for and is more centered in 8MR channels in zeolites than Na. Analogously, rubidium and water prevent distortion and stabilize the small, high-symmetry cell in Rb,Na-aluminogermanate JBW.^19^

## 5 Conclusion

In summary, large crystals of phase pure JBW-type zeolite were synthesized hydrothermally under moderate conditions. The structure was fully characterized via X-ray refinement, solid-state NMR spectroscopy, and electron microscopy in its as-made as well as de- and rehydrated states. NMR crystallography revealed defect free crystals with an exceptional degree of ordering of the T-atoms and sodium cations, leading to the observation of second-order quadrupolar NMR patterns for Al and Na and also distinctive resonances for different T-sites. This has never been observed for zeolites, rendering JBW a valuable reference material for developing modeling methods for NMR. JBW is a high-density, semicondensed framework containing little water (±4% in fully hydrated state). Yet, the structure changes significantly and irreversibly upon de- and rehydration, establishing an important structural role of water even in dense zeolites, impacting the pore geometry and, consequentially, properties like pore accessibility and diffusivity. These results hold implications for the transferability of molecular simulations of microporous materials to real-world applications.

## Supporting Information Available

Refined crystal structure of as-made and dehydrated JBW structures (CIF)
Diffraction patterns of rehydrated JBW, NMR spectra, chemical analysis, thermogravimetric analysis, and Rietveld refinement data as referenced in the manuscript (PDF)

## Supplementary Material

cm2c01059_si_001.cif
