# Supplementary material for: HSIL-Based Synthesis of Ultracrystalline K,Na-JBW, a Zeolite Exhibiting Exceptional Framework Ordering and Flexibility
Source: Chem Mater. 2022 Jun 16;34(16):7159–66. doi: 10.1021/acs.chemmater.2c01059 (PMC9404536; doi:10.1021/acs.chemmater.2c01059)
Supplement: Supplementary file 2 — cm2c01059_si_002.pdf [file cm2c01059_si_002.pdf]

Supporting info to:

HSIL based synthesis of ultra-crystalline K,Na-JBW, a zeolite exhibiting exceptional framework ordering and flexibility.

Karel Asselman[1], Sambhu Radhakrishnan[1,2], Nick Pellens[1], C. Vinod Chandran[1,2], Maarten Houlleberghs[1], Yijue Xu[3], Johan A. Martens[1], Sreeprasanth Pulinthanathu Sree[1], Christine E.A. Kirschhock[1], Eric Breynaert[1,2,3,*]

[1]Center for Surface Chemistry and Catalysis – Characterisation and Application Team (COK-KAT), KU Leuven, Celestijnenlaan 200F, 3000 Leuven, Belgium
[2]NMR-Xray platform for Convergence Research (NMRCoRe), KU Leuven, Celestijnenlaan 200F, 3000 Leuven, Belgium
[3]National High Magnetic Field Laboratory, 1860 East Paul Dirac Drive, Tallahassee, Florida 32310, United States

*Corresponding author

e-mail: eric.breynaert@kuleuven.be
Postal adress: Celestijnenlaan 200F, 3001 Leuven, Belgium

Table S1: Chemical analysis of as-made JBW

Si (ICP)	Al (ICP)	Na (AAS)	K (AAS)	H_2O (^{1}H-NMR)	Si/Al	Na/K
		wt%			ratio	ratio
18.52	17.91	10.10	7.44	4.04	0.995	2.31

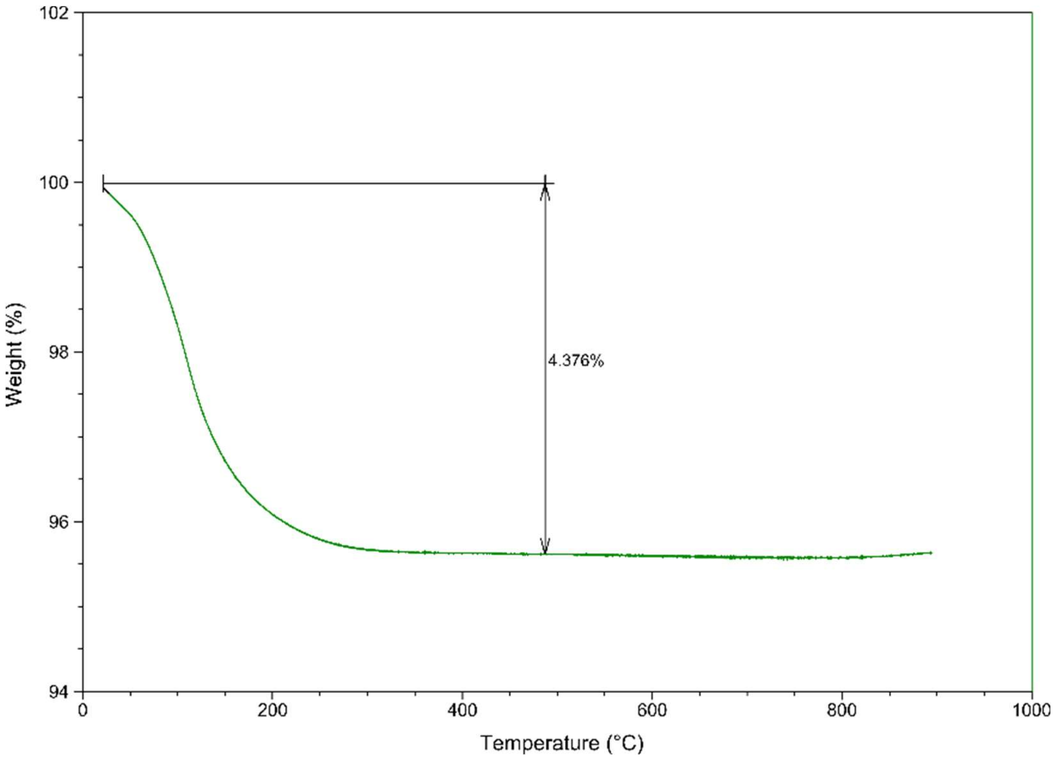

Figure S1: TGA profile of as-made JBW

Table S2: Structure parameters of as made JBW

Scatterer	*Mult.*	*Occupancy*	*x*	*y*	*Z*	U_{iso} *(Å^2)**
Si1	4	1	0.85810(22)	0.38588(34)	0.4384(6)	0.0144(11)
Si2	2	1	0	0.8843(5)	0.9386(8)	0.0111(15)
Al1	4	1	0.64431(24)	0.3984(4)	0.4504(6)	0.0271(14)
Al2	2	1	0	0.0984(5)	0.4347(9)	0.0176(18)
O1	2	1	0	0.0527(8)	0.1051(11)	0.0180(6)
O2	4	1	0.91513(32)	0.7780(5)	0.0272(11)	0.0180(6)
O3	4	1	0.75478(27)	0.3503(4)	0.4644(10)	0.0180(6)
O4	4	1	0.8852(4)	0.5225(7)	0.6491(9)	0.0180(6)
O5	4	1	0.8928(4)	0.4500(6)	0.1614(9)	0.0180(6)
O6	4	1	0.90666(28)	0.2148(5)	0.5053(12)	0.0180(6)
O7	2	1	0	0.9243(8)	0.6320(11)	0.0180(6)
Na1	2	1	0	0.3118(6)	0.9347(13)	0.0144(18)
Na2	2	1	0	0.6575(7)	0.3859(11)	0.0258(22)
K1	4	0.5	0.2439(7)	1.0490(5)	0.7477(9)	0.0637(20)
Ow1	4	0.469	0.2922(8)	1.0250(19)	0.6125(24)	0.092(7)

*displacement factors U_{iso} of framework oxygen were constrained to be equal

Table S3: Structure parameters of dehydrated JBW

Scatterer	*Mult.*	*Occupancy*	*x*	*y*	*z*	U_{iso} *(Å^2)^a*
Al1	2	1	0.1310(11)	0.2533(17)	0.4048(6)	0.0182(6)
Al2	2	1	0.0823(10)	0.2732(16)	0.1089(5)	0.0182(6)
Al3	2	1	0.5883(9)	0.7791(17)	0.2386(6)	0.0182(6)
Si1	2	1	0.8580(10)	0.7624(16)	0.0933(6)	0.0182(6)
Si2	2	1	0.8947(9)	0.7724(15)	0.3826(5)	0.0182(6)
Si3	2	1	0.6227(9)	0.7243(17)	0.7390(6)	0.0182(6)
O1	2	1	0.9220(16)	0.0577(22)	0.1180(10)	0.0183(10)
O2	2	1	0.0516(17)	0.5664(24)	0.3800(10)	0.0183(10)
O3	2	1	0.0239(16)	0.9440(23)	0.6504(9)	0.0183(10)
O4	2	1	1.0107(17)	0.4360(24)	0.8565(9)	0.0183(10)
O5	2	1	0.1760(17)	0.2799(32)	0.0092(7)	0.0183(10)
O6	2	1	0.5872(16)	0.4054(22)	0.7486(10)	0.0183(10)
O7	2	1	0.1521(18)	0.2095(31)	0.5131(7)	0.0183(10)
O8	2	1	0.2318(14)	0.2045(31)	0.1876(7)	0.0183(10)

O9	2	1	0.3239(16)	0.2423(29)	0.3617(8)	0.0183(10)
O10	2	1	0.7291(13)	0.6969(28)	0.3226(7)	0.0183(10)
O11	2	1	0.6846(16)	0.7104(27)	0.1421(8)	0.0183(10)
O12	2	1	0.5523(16)	0.1152(22)	0.2485(10)	0.0183(10)
K1	2	0.5	0.5131(21)	0.1080(26)	0.5064(11)	0.064(6)
K2	2	0.5	0.4801(21)	0.5985(25)	-0.0119(10)	0.046(6)
Na1	2	1	0.8322(15)	0.1996(21)	0.7485(7)	0.025(4)
Na2	2	1	0.1633(14)	0.7120(23)	0.7540(7)	0.029(4)

*displacement factors U_{iso} of framework oxygen and framework T-sites were respectively constrained to be equal

Table S4: Rietveld refinement parameters of as-made JBW

Wavelength (Å)	1.540598 (CuKα1)
Space group	$Pmn2_1$
2θ range (degree)	7.00 – 89.90
Resolution (Å)	1.09
Number of reflections	313
Lattice constants (Å):	
a	15.14326(8)
b	8.12637(6)
c	5.176559(30)
Unit cell volume (Å^3)	637.025(4)
Residuals:	
R_{wp}	0.0621
R_{F2}	0.0592

Table S5: Rietveld refinement parameters of dehydrated JBW

Wavelength (Å)	1.540598
Space group	P-1
2θ range (degree)	5.00-80.02
Resolution (Å)	1.199
Number of reflections	761
Lattice constants (Å):	
a	7.94578(12)
b	5.16870(6)
c	15.16537(20)
α	89.8480(16)
β	93.6133(9)
γ	89.9481(16)
Unit cell volume (Å^3)	621.591(17)
Residuals:	
R_{wp}	0.0906
R_{F2}	0.0999

Table S6: Bond lengths of as made JBW

	bond length (Å)		bond length (Å)
Si(1)-O(3)	1.597(8)	Al(1)-O(2)	1.746(6)
Si(1)-O(4)	1.578(7)	Al(1)-O(3)	1.712(8)
Si(1)-O(5)	1.605(7)	Al(1)-O(4)	1.784(7)
Si(1)-O(6)	1.605(6)	Al(1)-O(5)	1.745(6)
Si(2)-O(1)	1.611(10))	Al(2)-O(1)	1.769(10)
Si(2)-O(2)	1.608(6)	Al(2)-O(6)	1.737(6)
Si(2)-O(7)	1.615(9)	Al(2)-O(7)	1.739(9)
O(1)-Na(1)	2.309(10)	O(5)-Na(1)	2.303(8)
O(4)-Na(1)	2.854(8)	O(6)-Na(1)	2.776(8)
O(4)-Na(2)	2.462(8)	O(2)-Na(2)	2.437(7)
O(7)-Na(2)	2.481(10)	O(5)-Na(2)	2.599(7)

Table S7: Bond lengths of dehydrated JBW

	bond length (Å)		bond length (Å)
Si(1)-O(1)	1.646(9)	Al(3)-O(6)	1.711(10)
Si(1)-O(4)	1.616(9)	Al(3)-O(10)	1.695(10)
Si(1)-O(5)	1.578(9)	Al(3)-O(11)	1.732(11)
Si(1)-O(11)	1.627(9)	Al(3)-O(12)	1.769(11)
Si(2)-O(2)	1.641(9)	O(1)-Na(2)	2.413(18)
Si(2)-O(3)	1.690(9)	O(2)-Na(1)	2.516(18)
Si(2)-O(7)	1.650(9)	O(2)-Na(2)	2.949(18)
Si(2)-O(10)	1.601(9)	O(3)-Na(1)	2.563(19)
Si(3)-O(6)	1.680(9)	O(3)-Na(2)	2.214(16)
Si(3)-O(8)	1.598(9)	O(4)-Na(1)	2.430(16)
Si(3)-O(9)	1.619(9)	O(4)-Na(2)	2.480(19)
Si(3)-O(12)	1.640(9)	O(6)-Na(1)	2.218(17)
Al(1)-O(2)	1.768(11)	O(8)-Na(1)	2.369(18)
Al(1)-O(3)	1.770(10)		
Al(1)-O(7)	1.656(10)		
Al(1)-O(9)	1.704(10)		
Al(2)-O(1)	1.705(11)		
Al(2)-O(4)	1.769(11)		
Al(2)-O(5)	1.727(11)		
Al(2)-O(8)	1.667(10)		

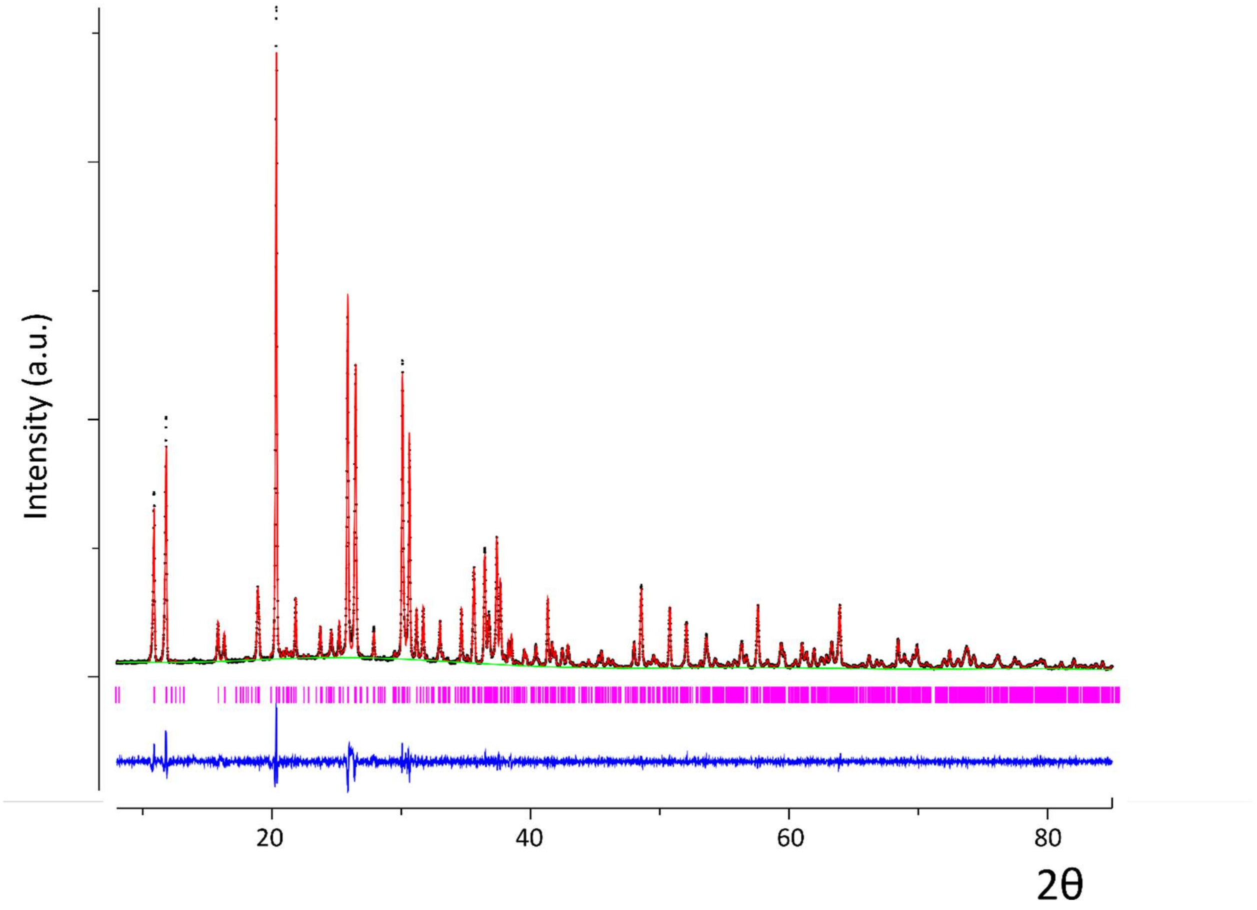

Fig. S2: Le Bail profile fit of rehydrated JBW. a = 16.29 Å, b = 5.18 Å, c = 15.00 Å, α = 89.90°, β = 91.74°, γ = 89.98° (Rwp = 0.0675).

Table S8: NMR parameters (^{29}Si, ^{27}Al & ^{23}Na) of as-made hydrated form and dehydrated JBW

Na,K-JBW form	^{29}Si			^{27}Al				^{23}Na			
	$\delta(^{29}Si)$ ppm	Width ppm	%	$\delta(^{27}Al)$ ppm	C_Q MHz	η_Q	%	$\delta(^{23}Na)$ ppm	C_Q MHz	η_Q	%
**As-made**	-84.0	1.2	34	63.7	1.8	0.69	33	7.9	1.1	0.62	43
	-87.0	1.8	66	61.7	3.2	0.39	67	5.8	1.0	0.47	44
								5.0	2.1	0.61	10
								1.9	2.8	0.29	3
**Dehydrated**	-83.5	1.3	34	65.0	1.7	0.7	33	10.5	1.2	0.58	48
	-85.2	1.5	31	63.8	2.7	0.4	34	7.7	1.0	0.54	48
	-87.2	1.6	35	61.1	4.0	0.5	33	-4.6	1.7	0.67	4

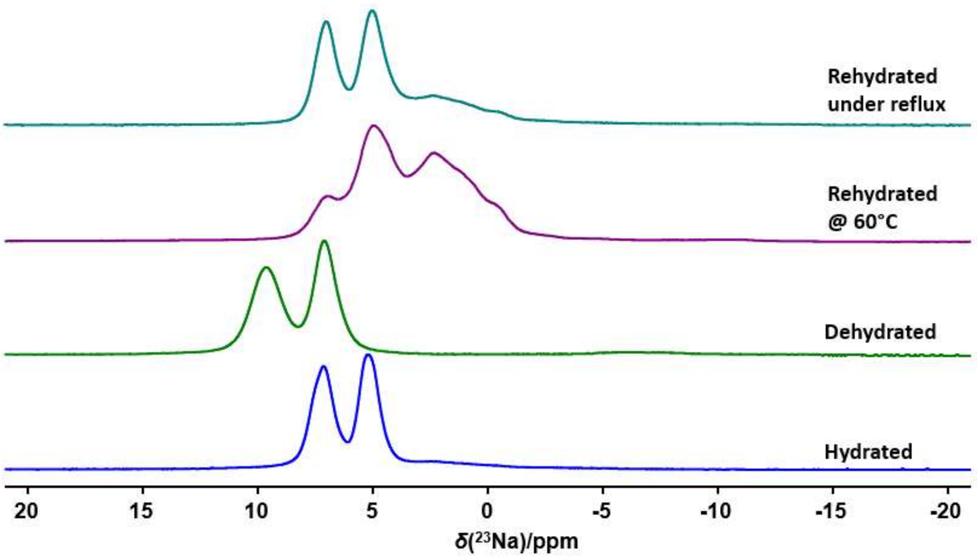

Fig. S3: ^{23}Na NMR spectra of JBW samples after various treatments. After refluxing, the spectrum shows a mixture of restored (as-made) and unrestored (rehydrated @60°C in a closed vessel prior to refluxing) phases.

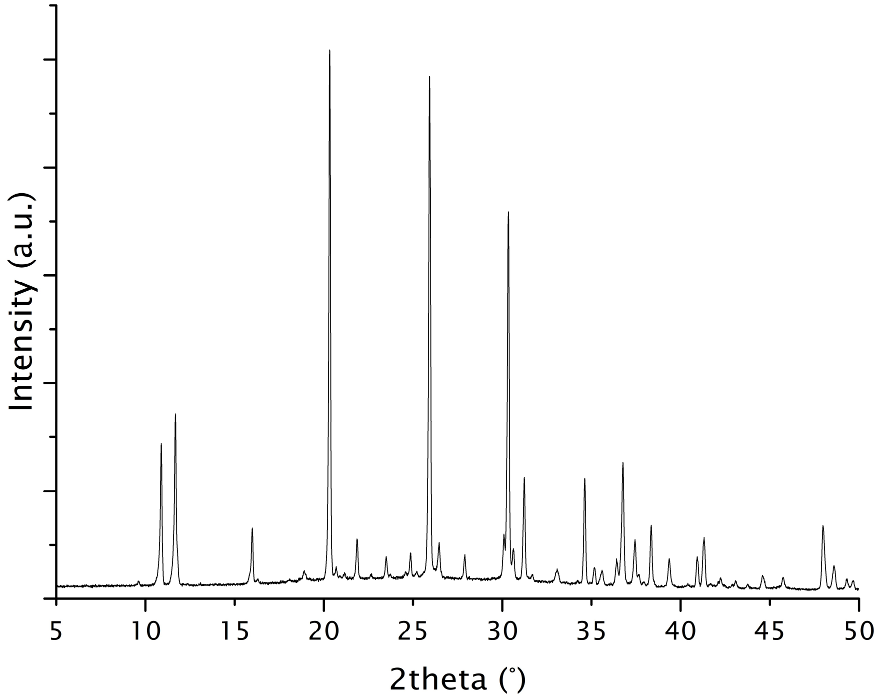

Fig. S4: Powder diffraction pattern of rehydrated JBW after refluxing. This time, the powder patterns show a mixture of both restored (identical to as made) and unrestored (rehydrated @60°C in a closed vessel prior to refluxing) phases.

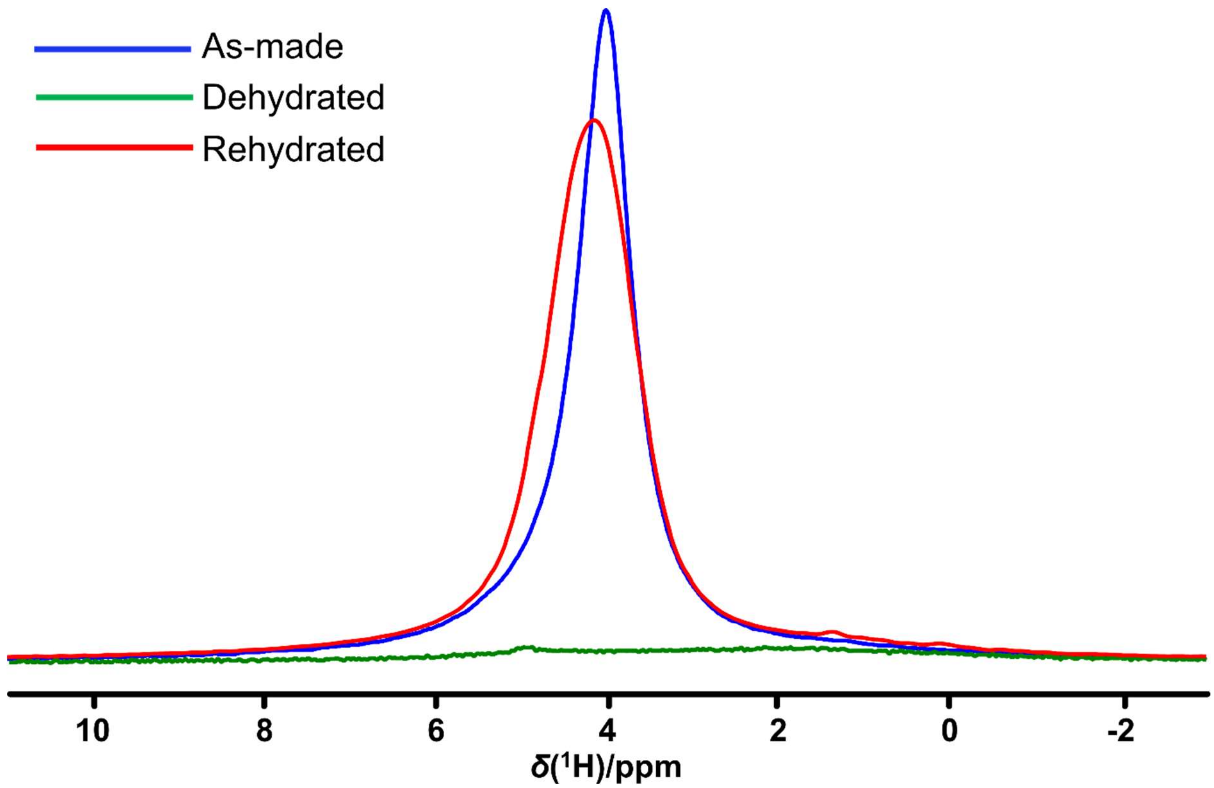

Fig. S5: Quantitative ^{1}H NMR spectra of as-made, de- and rehydrated JBW samples. After rehydration, the structure readily reabsorbs the original water content, but the spectrum indicates the water molecules have a broader distribution in the rehydrated phase.

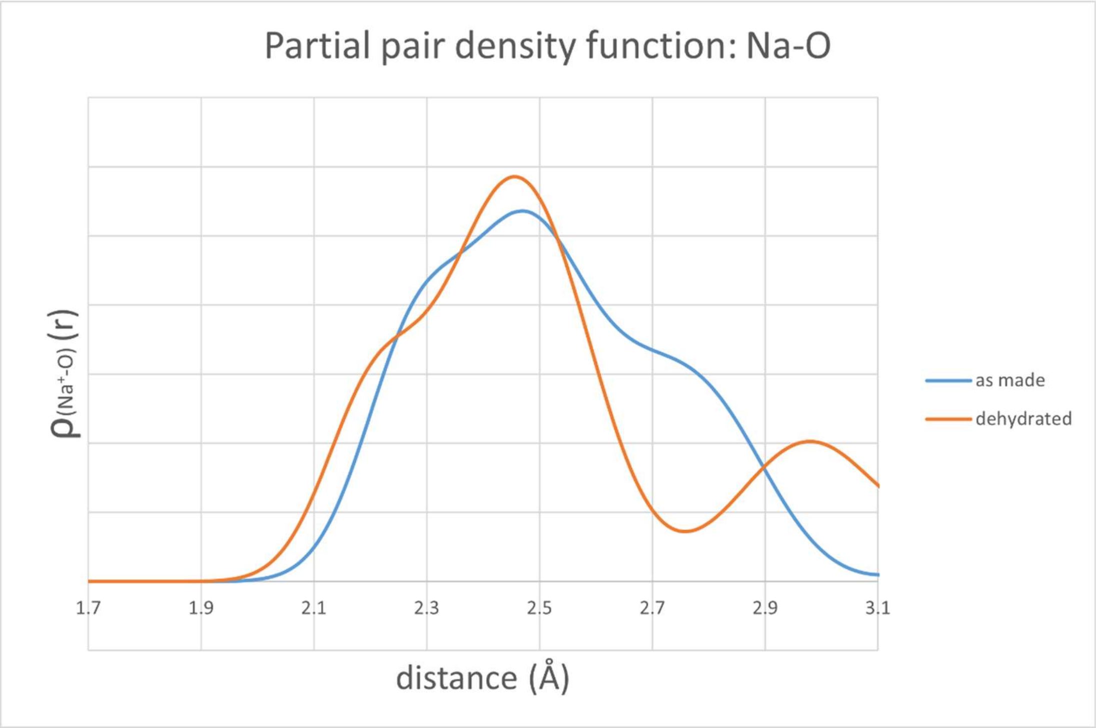

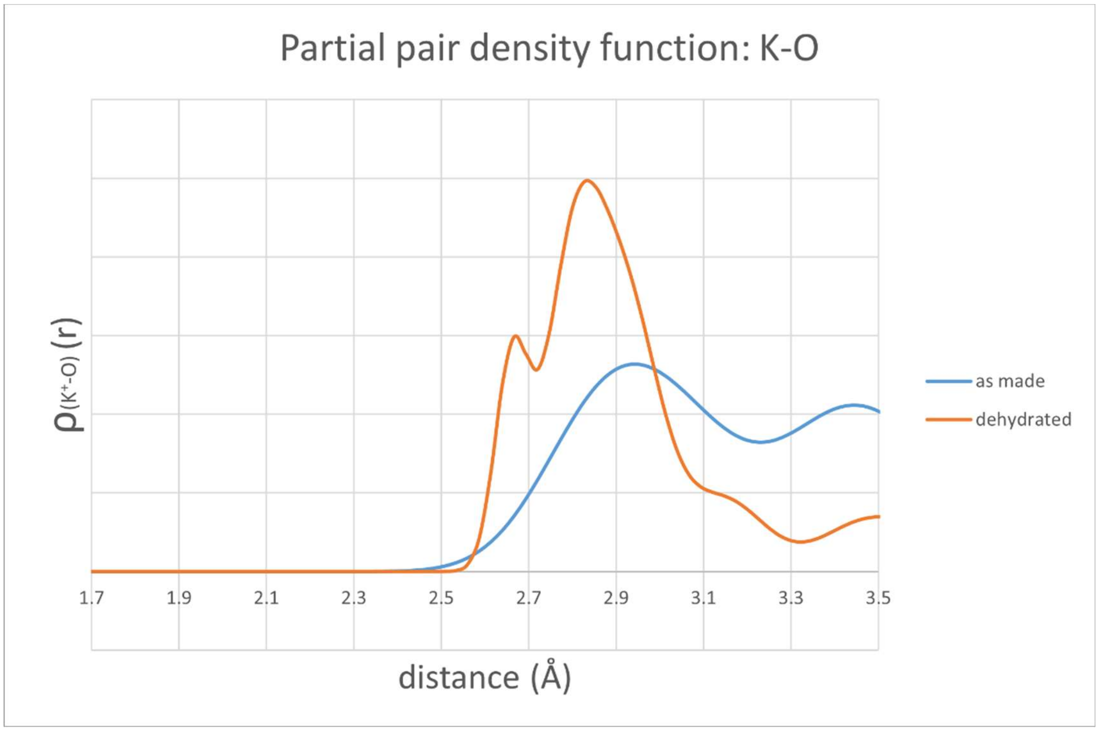

Fig. S6: Normalized partial pair density functions ($Å^{-3}$) for K and Na with framework oxygen, for as made and dehydrated JBW. The PDFs were calculated from the refined crystal structures in GULP[1,2].

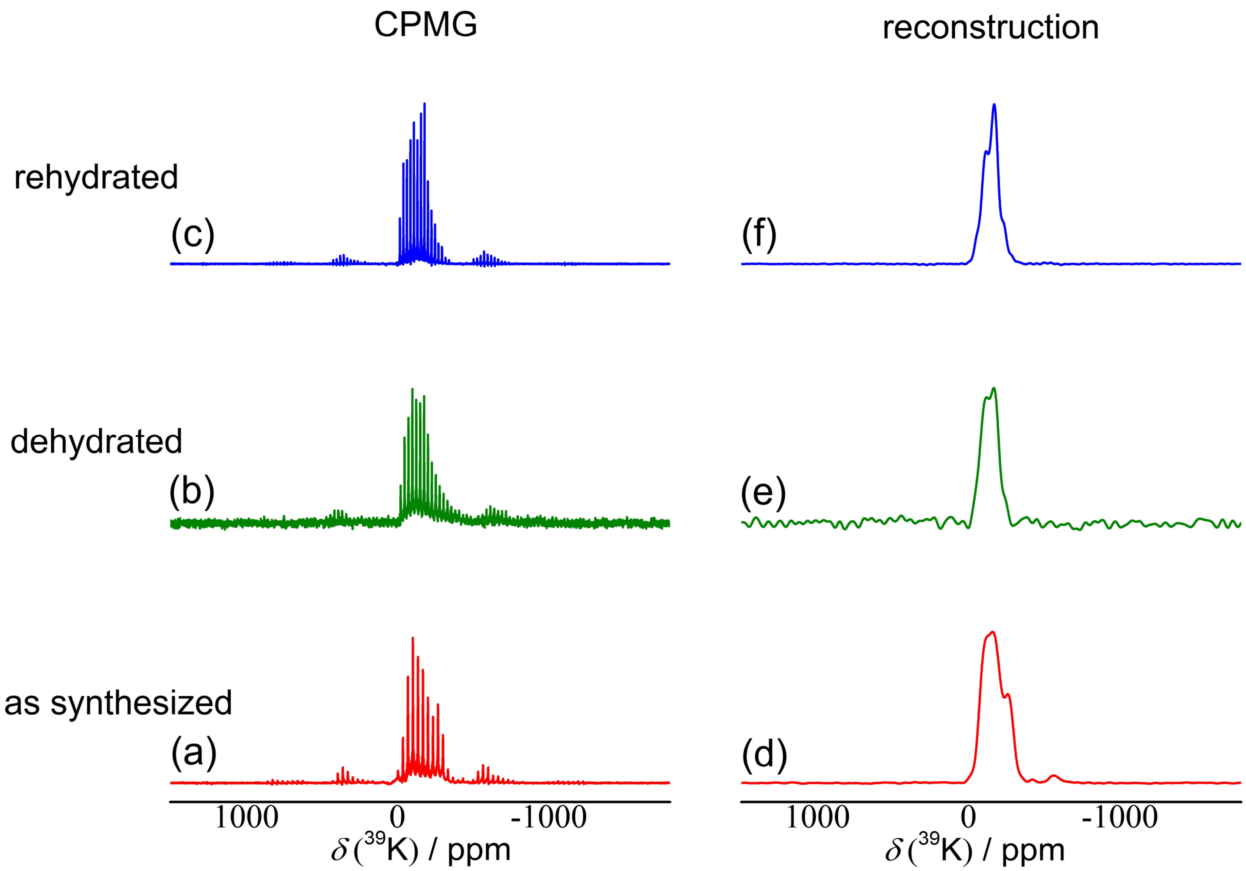

Fig. S7: ^{39}K spikelet patterns of JBW samples and their reconstructions.

References

(1) Gale, J. D.; Rohl, A. L. The General Utility Lattice Program (GULP). *Mol. Simul.* **2003**, *29* (5), 291–341.

(2) Cope, E. R.; Dove, M. T. Pair Distribution Functions Calculated from Interatomic Potential Models Using the General Utility Lattice Program. *J. Appl. Crystallogr.* **2007**, *40* (3), 589–594.
